# Amaryllidaceae alkaloids with anti-*Trypanosoma cruzi* activity

**DOI:** 10.1186/s13071-020-04171-6

**Published:** 2020-06-10

**Authors:** Nieves Martinez-Peinado, Nuria Cortes-Serra, Laura Torras-Claveria, Maria-Jesus Pinazo, Joaquim Gascon, Jaume Bastida, Julio Alonso-Padilla

**Affiliations:** 1 Barcelona Institute for Global Health (ISGlobal), Hospital Clínic - University of Barcelona, 08036 Barcelona, Spain; 2 Departament de Biologia, Sanitat i Medi Ambient, Facultat de Farmàcia i Ciències de l´Alimentació, Universitat de Barcelona, 08028 Barcelona, Spain

**Keywords:** Chagas disease, *Trypanosoma cruzi*, Alkaloids, Amaryllidaceae, Hippeastrine, Phenotypic assays, Cytotoxicity

## Abstract

**Background:**

Chagas disease, caused by the protozoan *Trypanosoma cruzi*, is a neglected disease that affects ~7 million people worldwide. Development of new drugs to treat the infection remains a priority since those currently available have frequent side effects and limited efficacy at the chronic stage. Natural products provide a pool of diversity structures to lead the chemical synthesis of novel molecules for this purpose. Herein we analyzed the anti-*T. cruzi* activity of nine alkaloids derived from plants of the family Amaryllidaceae.

**Methods:**

The activity of each alkaloid was assessed by means of an anti-*T. cruzi* phenotypic assay. We further evaluated the compounds that inhibited parasite growth on two distinct cytotoxicity assays to discard those that were toxic to host cells and assure parasite selectivity.

**Results:**

We identified a single compound (hippeastrine) that was selectively active against the parasite yielding selectivity indexes of 12.7 and 35.2 against Vero and HepG2 cells, respectively. Moreover, it showed specific activity against the amastigote stage (IC_50_ = 3.31 μM).

**Conclusions:**

Results reported here suggest that natural products are an interesting source of new compounds for the development of drugs against Chagas disease.
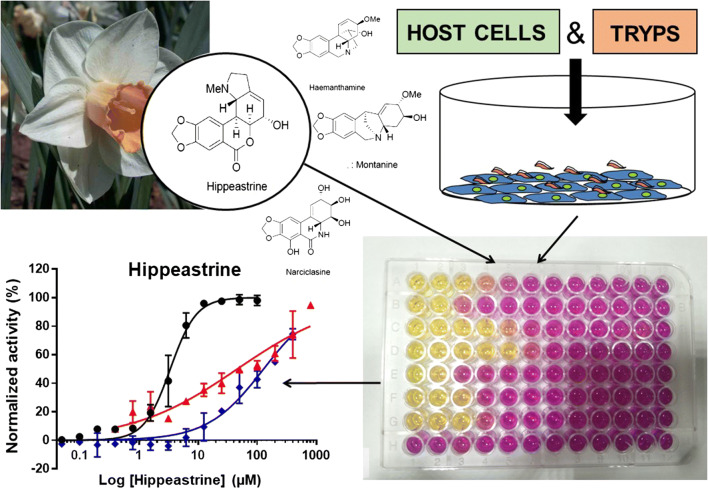

## Background

Chagas disease (or American trypanosomiasis) is a neglected infectious disease caused by the protozoan parasite *Trypanosoma cruzi* (order Kinetoplastida; family Trypanosomatidae). It is estimated that ~7 million people are affected by the disease, mainly in Latin America where *T. cruzi* infection is endemic [1].

The disease progresses in two phases. There is first a short acute phase that is usually asymptomatic and thus goes unnoticed. This is followed by a chronic phase characterized by absent or slow progression of clinical manifestations [2]. Nonetheless, it is estimated that ~40% of those chronically infected will ultimately develop disruptive damage to the heart and/or digestive tract (esophagus and colon) tissues, which can lead to the formation of mega-syndromes and death if untreated [2, 3].

Since the 1970s only two drugs have been available to treat *T. cruzi* infections: benznidazole (BNZ) and nifurtimox (NFX) [1]. Both have good efficacy and tolerability when administered to infected new-borns [4]. But their efficacy diminishes at the chronic stage, which is usually diagnosed at adulthood with serological tests that detect specific anti-*T. cruzi* type G immunoglobulins [1]. Moreover, both drugs have long regimens of administration that entail the advent of frequent adverse events which often drive to treatment discontinuation [5–7]. There is thus an urgent unmet need of safer and more efficacious drugs for the treatment of chronic Chagas disease, for which natural products may represent a promising approach to discover new lead compounds [8–10].

In this regard, members of the family Amaryllidaceae have attracted considerable attention in the last few years due to their unique alkaloid composition with multiple biological activities [11]. Amaryllidaceae plants have been studied for their potential application as a source of anticancer, anti-inflammatory, antimicrobial, anti-parasitic and anticholinesterase activities [12]. In fact, they have been used for centuries as part of traditional treatments for fever, swelling, cancer or malaria [12]. Remarkably, in 2001 the Food and Drug Administration (FDA) approved the use of galanthamine (trade name Razadyne), an alkaloid identified from the Amaryllidaceae plant *Galanthus woronowi*, to treat Alzheimer’s disease [13].

**Fig. 1:**
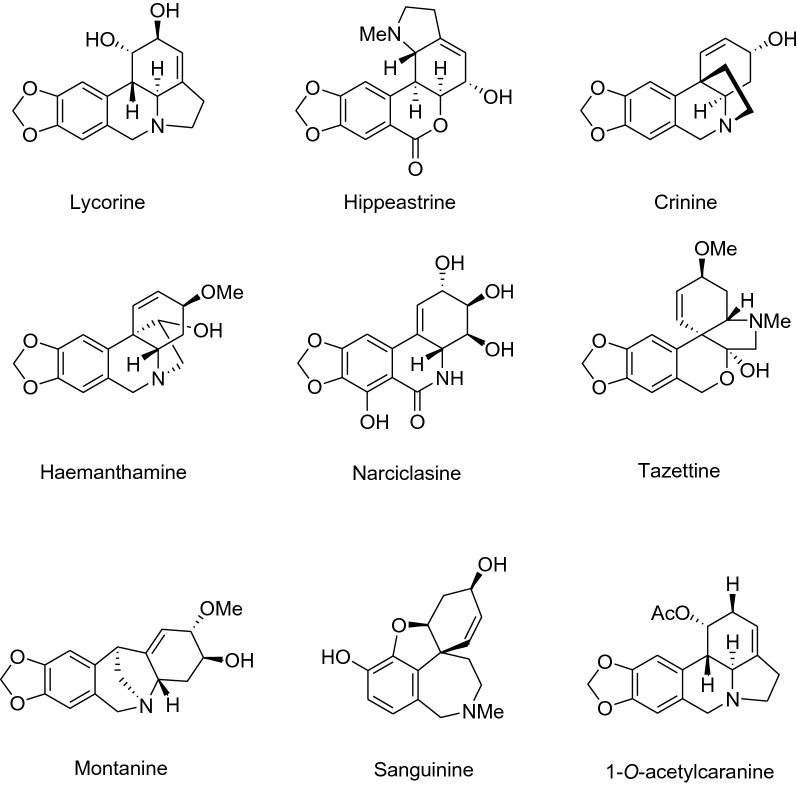
Chemical structures of the alkaloids evaluated in this study

Alkaloid constituents found in these plants are classified in eight groups based on structure and biogenesis from the common precursor *O*-methylnorbelladine: galanthamine; lycorine; crinine; haemanthamine; homolycorine; narciclasine; tazettine; and montanine [14]. The unique structure of this set of alkaloids provides a viable platform for phytochemical‐based drug discovery [8]. With the aim to identify prospective drug development starting points that could eventually become new therapeutic solutions for Chagas disease we have adapted an *in vitro* anti-*T. cruzi* phenotypic assay based on the parasite Tulahuen strain engineered to express a bacterial β-galactosidase gene [15] and green monkey epithelial cells (Vero) as hosts. We evaluated the anti-*T. cruzi* activity of nine crystalized alkaloid compounds extracted from members of the Amaryllidaceae family: lycorine, hippeastrine, crinine, haemanthamine, narciclasine, tazettine, montanine, sanguinine and 1-*O*-acetylcaranine (Fig. 1) [16]. In all the assays performed we always included the standard anti-parasitic drug BNZ for comparison.

In order to unveil the specific *T. cruzi* growth inhibitory capacity of those compounds that were found active in the anti-parasitic assay, we further used two secondary biological assays to determine the compounds’ level of cytotoxicity. These were respectively based on the same host Vero cells and in the human hepatocellular carcinoma cell line HepG2. Finally, we determined the anti-amastigote specific activity of the only compound that was revealed to hold selective anti-parasitic activity. Results obtained were particularly promising for the compound hippeastrine from *Narcissus* cv. Salome [17] and they are discussed herein.

## Methods

### Collection of purified alkaloid compounds from Amaryllidaceae plants

Lycorine, hippeastrine, crinine, haemanthamine, narciclasine, tazettine, montanine, sanguinine and 1-*O*-acetylcaranine alkaloids were isolated from extracts of different *Narcissus* species [16, 18]. The information of all the compounds studied can be found in the extensive chapter by Bastida et al. [16]. In brief, the procedure followed to identify the alkaloids within the corresponding plant extract was as follows: plant material (60 mg) was macerated with MeOH; the mix was filtered and the solvent evaporated to dryness. After that, extracts were acidified with 500 µl of H_2_SO_4_ (2%, v/v). The neutral material was removed with diethyl ether and basified with 200 µl NH_4_OH (25 %, v/v). Then, 750 µl of diethyl ether was added to separate the organic phase, this was repeated twice, and the solvent evaporated to dryness. All compounds were crystals, obtained after three successive crystallization rounds to ensure maximum purity. They were subjected to a combination of chromatographic techniques and alkaloids were identified by GC-MS and NMR [16, 17] (Additional file 1: Figures S1, S2). In order to obtain milligrams of product, the corresponding scale-up was performed as previously described [19, 20].

### Host cells cultures

Vero (green monkey kidney epithelial cells), LLC-MK2 (Reshus monkey kidney epithelial cells) and HepG2 (human liver epithelial cells) cultures were maintained with DMEM supplemented with 1% penicillin-streptomycin (100 units/ml of penicillin and 100 µg/ml of streptomycin; P-S) and 10% heat inactivated fetal bovine serum (FBS) at 37 °C, 5% CO_2_ and > 95% humidity as described by Buckner et al. [15]. HepG2 were also supplemented with 1× non-essential amino acids (ref. 01-340-1B; Biological Industries, Beit-Haemek, Israel).

### Culture of *T. cruzi* parasites

*Trypanosoma cruzi* parasites from the Tulahuen strain (discrete typing unit, DTU VI) expressing β-galactosidase were kindly provided by Dr Fred Buckner (University of Washington, Seattle, USA) and maintained using LLC-MK2 cells as hosts in DMEM supplemented with 2% FBS and 1% P-S as previously described [15]. Free-swimming trypomastigotes were purified by centrifugation of the cell culture supernatant for 7 min at 2500× *rpm* using a low break speed, then allowing them to swim out of the pellet [21]. Purified trypomastigotes were used to keep the parasite cycle in LLC-MK2 cells and for the performance of the anti-parasitic assays. In the last case, an extra round of centrifugation was performed to remove phenol red from the maintenance DMEM and replace with a phenol red-free DMEM medium, which was supplemented with 1% P-S-glutamine, 2% FBS, 1 mM sodium-pyruvate and 25 mM HEPES [21].

### Assay to detect *T. cruzi* growth inhibition in 96-well plates

Our assay is based on Vero cells as hosts and infective trypomastigotes from the Tulahuen strain that express the bacterial β-galactosidase enzyme as reporter activity [15]. First, alkaloids were added in the first column of a 96-well tissue culture treated plates at an initial concentration of 100 µM and diluted in assay medium into the next columns of the plate to conform dose-response plate-maps following either a 1:2 or 1:3 fold pattern. Then, Vero cells were detached from their growing flasks, counted and diluted at a concentration of 1 × 10^6^ cells per ml. Trypan blue staining was used to check their viability, which had to be > 95% to proceed. In conjunction, purified trypomastigotes were counted and diluted at a concentration of 1 × 10^6^ cells per ml. We directly mixed Vero cells and trypomastigotes in a falcon tube in a sufficient volume so as to add 100 μl of the mix per well (50,000 Vero cells and 50,000 trypomastigote cells per well; with the multiplicity of infection, MOI = 1). The percentage of DMSO in all wells was always kept below 0.5%.

The reference drug BNZ was used as a control of drug growth inhibition in each run, whereas each plate contained its own negative (maximum parasite growth; Vero cells plus parasites without drugs) and positive (minimum parasite growth; trypomastigote forms alone marking an enzymatic zero time or baseline galactosidase activity) controls. Note that trypomastigotes are unable to multiply in the absence of susceptible host cells. Plates were incubated for 4 days at 37 °C [21], with the readout performed by adding 50 µl per well of a PBS solution containing 0.25% NP40 and 500 µM chlorophenol red-β-d-galactoside (CPRG) substrate, as previously described [21]. Upon addition of the substrate, plates were further incubated at 37 °C for another 4 h and the absorbance read out at 590 nm using an Epoch Gene5 spectrophotometer (Biotek, Winooski, USA). All experiments were performed at least in triplicate.

### Anti-amastigote specific activity of progressed compound (hippeastrine)

Using Vero cells as hosts and the recombinant *T. cruzi* strain expressing β-galactosidase, we further adapted the anti-parasitic assay described above to determine whether the anti-parasitic activity was specific against the intracellular amastigote forms. In brief, we plated 50,000 Vero cells per well in a 96-well plate and allowed them to attach for 1.5 h. Then, we infected the monolayers with 50,000 purified trypomastigotes per well (MOI = 1) that were allowed for 1 h to adsorb and enter the cells before being washed with PBS three times. Finally, assay medium was added and used to dilute hippeastrine and BNZ in a dose-response pattern. In each plate we included the same controls as for the anti-*T. cruzi* assay. Test plates were incubated for 96 h and the assay readout was performed as described above.

### Toxicity assays with Vero and HepG2 cells

For the cell toxicity assays, compounds were added to tissue culture treated 96-well plates following a dose-response dilution pattern, 1:2 or 1:3, with a starting concentration of 400 µM or 800 µM per well in the first column of the plate. Cell viability was checked upon cell counting with Trypan blue and we only proceeded if viability was > 95%. Vero cell suspension was diluted at a concentration of 5 × 10^5^ cells per ml before adding 100 µl per well. In the case of HepG2 cells, we used a dilution of 3.2 × 10^4^ cells per ml. Each test plate or run contained its own negative (untreated cells) and positive (medium alone) controls. Plates were incubated at 37 °C for 4 days in the case of Vero cells, and 2 days for HepG2 cells. We then added 50 µl per well of a PBS solution containing 10% Alamar Blue reagent (Thermo Fisher Scientific, Eugene, USA) and incubated the plates for 6 h at 37 °C before reading the fluorescence intensity in a Tecan Infinite M Nano^+^ reader (Tecan, Männedorf, Switzerland) (excitation: 530 nm, emission: 590 nm). The percentage of DMSO in all wells was always kept below 0.5%. All experiments were performed at least in triplicate.

### Data analysis

Absorbance and fluorescence values derived from the anti-*T. cruzi* and cell toxicity assays were normalized to the controls [22]. IC_50_ and TC_50_ values were determined using GraphPad Prism 7 software (version 7.00, 2016) using a non-linear regression analysis model defined by the equation:

$$Y = 100 \div \left( {\left( {1 + X^{HillSlope} } \right) \div \left( {IC50^{HillSlope} } \right)} \right)$$

These IC_50_ and TC_50_ values are the compound concentrations capable of inhibiting growth of parasites and cells by 50%, respectively. *Z’-*values were calculated as described previously [23]. Values provided are the mean and standard deviation (SD) of at least three independent experiments.

## Results and discussion

### Quality assessment of the anti-*T. cruzi* and cell toxicity assays

**Fig. 2:**
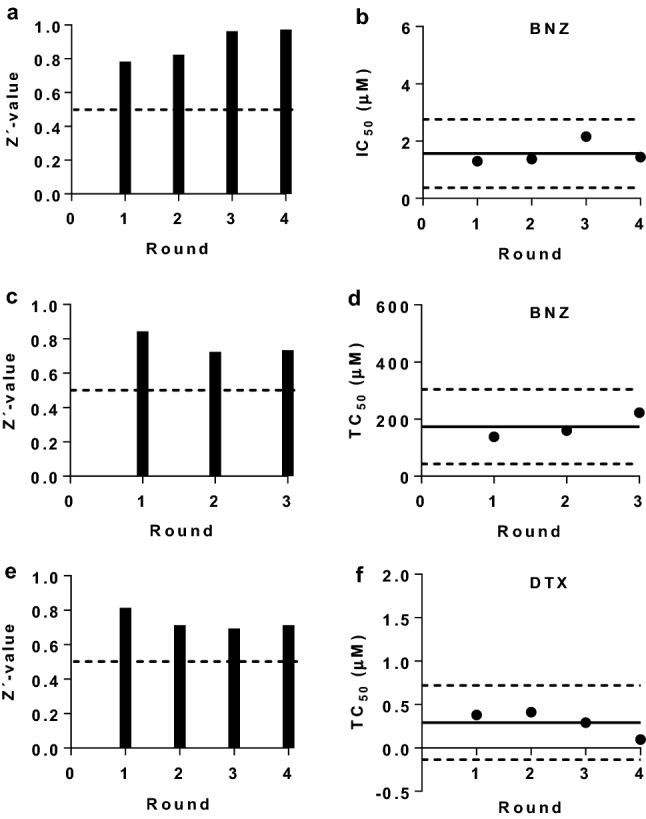
Quality controls of the *T. cruzi* growth inhibition assay (**a**, **b**), toxicity assay with Vero cells (**c**, **d**) and toxicity assay with HepG2 cells (**e**, **f**). *Z’-*values for each of the rounds launched are represented on the left (**a**, **c**, **e**); dashed line marks the 0.5 threshold. IC_50_ and TC_50_ values of the reference drugs BNZ and DTX are represented on the right (**b**, **d**, **f**); continuous lines indicate the average values, whereas the dashed lines indicate ± 3 SD limits

As part of the process of setting up the biological assays we calculated their *Z’* parameter to assess reproducibility and statistical robustness [23]. In general, assays with a *Z’* between 0.5 and 1 are considered appropriate for the screening of compounds [23]. Remarkably, our anti-parasitic assay had a very good performance and its *Z’-*value remained consistently > 0.5 with an average value of 0.89 (0.097) (Fig. 2a). Regarding the two cytotoxicity assays used in this study, we retrieved *Z’* = 0.76 (0.067) for the assay based on Vero cells, and a *Z’* = 0.73 (0.054) for the assay based on HepG2 cells (Fig. 2c–e).

Additionally, in every run of the *T. cruzi* growth inhibition assay and Vero cell toxicity assay performed, we included the reference drug BNZ as a control, whereas the reference drug digitoxin (DTX) [24] was included in all the HepG2 cells toxicity assays. Overall, averaged IC_50_ and TC_50_ values for BNZ were 1.56 (0.39) μM and 173.4 (43.57) μM, respectively (Fig. 2b–d, Additional file 1: Figure S3), which correlates with previous reports [21, 25]. The digitoxin TC_50_ mean value in the HepG2 cell assay was 0.29 (0.14) μM (Fig. 2f, Additional file 1: Figure S3).

### Anti-*T. cruzi* activity of the alkaloids extracted from Amaryllidaceae

As in other widely used anti-*T. cruzi* assays [21, 22, 26] we relied on the genetically robust *T. cruzi* Tulahuen strain expressing beta-galactosidase activity as a surrogate of parasite growth [15]. However, because the amastigote replicative stage of *T. cruzi* is obligatory intracellular, our assay relied on Vero cells as hosts. Notably, Vero cells have been described to be deficient in interferon response due to a mutation in the simian interferon beta gene [27], which makes them more susceptible to infection and thereby a good system for the discovery of active compounds against *T. cruzi*.

**Fig. 3:**
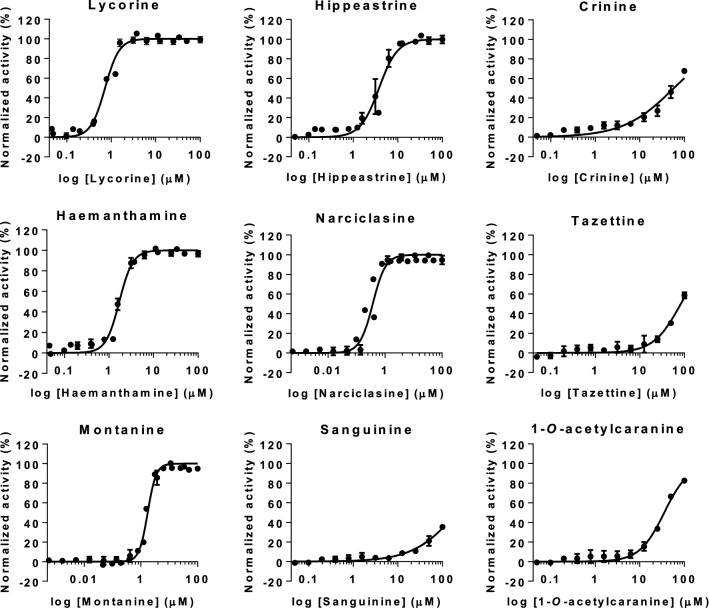
Anti-*T.cruzi* phenotypic assay dose-response curves. Graphs represent mean results and SD of at least three biological replicates

Results obtained from the phenotypic *T. cruzi* growth inhibition assay revealed that lycorine, hippeastrine, haemanthamine, narciclasine and montanine were active, while crinine, tazettine, sanguinine and 1-*O*-acetylcaranine were inactive against the parasite (Fig. 3). Tazettine, sanguinine and 1-*O*-acetylcaranine have been described as poor antiprotozoal agents before [28, 29]. Nonetheless, Machocho et al. [28] described that compound 3-*O*-acetylsanguinine had some activity (IC_50_ = 2.3 μg/ml; i.e. 7.29 μM) against trypomastigotes from the *T. cruzi* strain Tulahuen C4. Although different phenotypic anti-*T. cruzi* assays were performed in each case, the results obtained by these authors could suggest that the presence of an acetyl group might increase the anti-*T. cruzi* activity of sanguinine.

On the other hand, crinine and haemanthamine are crinane-type alkaloids that belong to the β-crinane and α-crinane subgroups, respectively [30]. Other studies evaluating the anti-parasitic potential of alkaloids have reported that the presence of a methylene-dioxi group seemed to favour a more potent anti-parasitic activity [31]. This could be the explanation for the different anti-*T. cruzi* activities observed by us between these two crinane-type alkaloids.

The highest anti-*T. cruzi* activity rates were yielded by lycorine [IC_50_ = 0.70 (0.018) µM] and narciclassine [IC_50_ = 0.495 (0.018) µM], which exceeded in potency that of the reference drug BNZ (Table 1, Additional file 1: Figure S3). Lycorine was the first alkaloid described from the plant family Amaryllidaceae and one of the most commonly found amongst genera in the family [32]. Lycorine extracted from *Crinum stuhlmannii*, *Zhepyranthes citrina* and *Narcissus broussonetii* has previously been tested against *T. cruzi* [33–35]. However, in contrast to our results, it was described as inactive against the parasite; a feature that could be explained due to the use of different assays. It has been reported that major differences in the activity of compounds can be found depending on the host cell line and the biological assay used [36]. In this respect, previous studies relied on the use of L6 cells as a host, whereas we have used Vero cells. Lycorine and 1-*O*-acetylcaranine belong to the lycorine group but structurally differ in two positions of the overall structure (Fig. 1). In this case, the anti-parasitic activity is increased with the substitution of a hydrogen and an acetoxy group per two hydroxyl groups.

**Table 1:** Alkaloid average IC_50_, TC_50_ and SI values for Vero and HepG2 cells

Alkaloid	IC_50_ (µM)	TC_50_ (µM)^a^	SI^a^	TC _50_ (µM)^b^	SI^b^
BNZ	1.56	173.4	111.15	168.76	108.18
Lycorine	0.70	5.21	7.44	21.87	31.24
Hippeastrine^c^	3.63	45.99	12.67	128.10	35.29
Crinine	57.93	–		–	
Haemanthamine	1.59	11.52	7.25	42.48	26.72
Narciclasine	0.49	0.66	1.33	2.73	5.52
Tazettine	83.03	–		–	
Montanine	1.99	5.04	2.53	46.10	23.17
Sanguinine	213.40	–		–	
1-*O*-acetylcaranine	35.49	–		–	

^a^Vero cell toxicity assay

^b^HepG2 cell toxicity assay

^c^The only alkaloid evaluated in this study that showed specific anti-*T. cruzi* activity

*Note*: The standard drug BNZ is included in the first line for comparison

Narciclasine yielded the most potent activity against *T. cruzi* in this study (Table 1). To the best of our knowledge, this compound has not been tested against *T. cruzi* before. Likewise, there are no previous reports on the anti-*T. cruzi* activity of montanine, also shown for the first time in this study. Montanine and haemanthamine showed average IC_50_ values similar to that of BNZ, respectively 1.99 (0.089) µM and 1.59 (0.062) µM *versus* 1.56 (0.070) µM of BNZ (Table 1. Additional file 1: Figure S3). Osorio et al. [29] have previously described the high activity of haemanthamine against *T. cruzi* (IC_50_ = 1.8 µg/ml; i.e. 5.97 μM).

Finally, we found that hippeastrine was the active alkaloid with a higher IC_50_ value [IC_50_ = 3.63 (0.24) µM]. Its activity was lower than that of BNZ, but still fell within a range five times the IC_50_ of the standard drug (Table 1, Additional file 1: Figure S3). Hippeastrine was first isolated from the Amaryllidaceae plant *Lycoris radiata* and has been reported to exhibit activity against avian influenza virus H5N1 [IC_50_ = 47.5 (0.37) µM] [37]. Moreover, promising results against ZIKA virus infection have recently been reported with hippeastrine hydrobromide [38]. This was shown to remove ZIKA virus from infected human neural progenitors, recover a ZIKV-induced microcephaly phenotype in human forebrain organoids and even suppress virus propagation in infected adult mice [38]. Antiviral [37, 38], antibacterial and antifungal [39] activities have been reported for hippeastrine, despite this, little information is available about its anti-parasitic activity. Cedron et al. [40] tested 21 hippeastrine derivatives that included functional group transformations, structural simplification and dimer formation against *Plasmodium falciparum* (strain F-32 Tanzania). The anti-malarial activity increased by 10-fold when dimers were evaluated compared to the single alkaloid activity, suggesting an improved binding to the related target or the hydrolysis of the dimer onto two molecules [40]. To our knowledge, this is the first time that anti-*T. cruzi* activity is reported for hippeastrine. Results reported by Cedron et al. [40] would suggest to further pursue research with hippeastrine derivatives against *T. cruzi*.

### Identification of alkaloid compounds with specific anti-*T. cruzi* activity

With the aim of further selecting those alkaloids with specific activity against the parasite and discard those that were toxic to host cells, we used two secondary cell toxicity assays with monkey (Vero) and human (HepG2) cells. Since the compounds activity might vary depending on the characteristics of the cell line used, performing the cytotoxicity assay in two cell lines will provide a more robust readout. Moreover, HepG2 cells are a widespread cellular model used to anticipate potential liver toxicity of drug metabolism [41, 42]. We determined a selectivity index (SI; or TC_50_ to IC_50_ ratio) > 10 to consider whether an alkaloid was suitable for further progression, as described elsewhere [22].

**Fig. 4:**
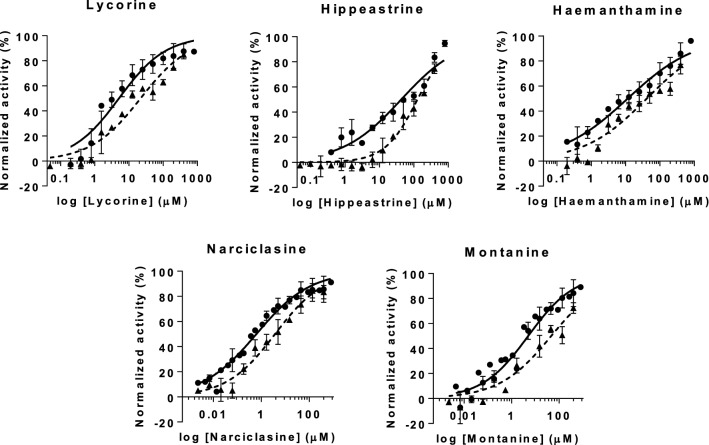
Dose-response curves obtained from the Vero and HepG2 cell toxicity assays. Vero cells toxicity assays are represented by circles and straight lines while HepG2 cell toxicity assays are represented by triangles and dashed lines. Graphs represent mean results and SD of at least three biological replicates

Thereafter, all the alkaloids reported as active against *T. cruzi* were analysed through both cell toxicity assays. All of them were more toxic to Vero cells than to HepG2 cells (Fig. 4). The cytotoxicity values registered against Vero and HepG2 cells indicated that narciclasine activity was not specific against *T. cruzi*, as it respectively showed TC_50_ = 0.66 (0.082) µM against Vero cells, and TC_50_ = 2.73 (0.67) µM against HepG2 cells, resulting in a SI < 10 in both cases (Table 1). In addition, montanine with a SI = 2.53 in relation to Vero cells was not specific to *T. cruzi* either (Table 1), even though it showed low toxicity to HepG2 cells [TC_50_ = 46.1 (11.99) µM] (Table 1). Something similar occurred with lycorine, which was weakly active against HepG2 cells [TC_50_ = 21.87 (4.16) µM] (Table 1). Lycorine presented a SI *versus* this cell line over ten times its registered anti-*T. cruzi* activity, but turned to be toxic to Vero cells [TC_50_ = 5.21 (0.80) µM] with a SI < 10 and thus was discarded from further progression (Table 1). We generally observed an increase sensitivity of Vero cells to the alkaloids when compared to HepG2 cells. This may have been in part due to the fact that compounds were incubated for a longer time (four *versus* two days) on Vero cells than on HepG2 cells.

Haemanthamine has been reported to present a TC_50_ = 13 µg/ml, i.e. 43.14 µM for HepG2 cells [43], which correlates with the TC_50_ value obtained in our study with the same cell line [TC_50_ = 42.48 (6.96) µM] (Table 1). However, although this compound showed a SI > 10 with respect to HepG2 cells, when evaluated on Vero cells its TC_50_ to IC_50_ ratio was below that threshold and thus its anti-parasitic activity could not be considered specific (Table 1).

**Fig. 5:**
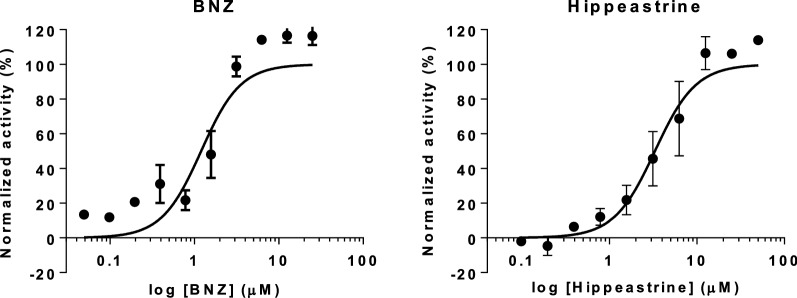
Anti-amastigote dose response curves of hippeastrine and BNZ. Graphs represent mean results and SD of at least three replicates

In contrast to all the aforementioned results, hippeastrine did show low toxicity against Vero cells [TC_50_ = 45.99 (6.32) µM] and HepG2 cells [TC_50_ = 128.1 (12.26) µM], and complied with the SI window > 10 against both cell lines (Table 1). It was the only compound that had a SI > 10 *versus* Vero cells (SI = 12.67; Table 1). Additionally, hippeastrine presented the highest SI against HepG2 cells (SI = 35.29), with a TC_50_ value similar to that previously reported by Weniger et al. [43] (TC_50_ = 40 µg/ml, i.e. 126.85 µM). Therefore, in a subsequent anti-amastigote biological assay we assessed whether hippeastrine anti-*T. cruzi* activity was indeed specific against this replicative form of the parasite. We found that the observed anti-amastigote activity (IC_50_) was 3.31 (0.39) µM, which was again within 5× that of the reference drug BNZ in the same assay [IC_50_ = 1.2 (0.22) µM] (Fig. 5). Moreover, the corresponding SI for hippeastrine with respect to its anti-amastigote activity will yet be > 10 (SI = 13.89 against Vero cells, and 38.69 against HepG2 cells).

Cytotoxicity results together with *in vivo* assays reported by Zhou et al. [38] may predict a low toxicity of hippeastrine in a future evaluation of its anti-*T. cruzi* activity in animal models. However, before performing *in vivo* studies, additional *in vitro* studies should be pursued to better validate this alkaloid. For example, assessing its effect on a *T. cruzi* CYP51 target [44], since this drug target has been invalidated in the clinic [45, 46], and identifying whether it can kill dormant parasite forms [47]. Moreover, it would be of interest to determine key *in vitro* pharmacokinetic (PK) parameters such as its solubility, permeability and clearance.

## Conclusions

We identified one compound with specific anti-*T. cruzi* activity, upon the evaluation of nine alkaloids purified from extracts of different *Narcissus* species (family Amaryllidaceae) [16], proving that natural products are an interesting source to potentially identify new chemical structures for Chagas disease drug discovery. Our findings suggest that hippeastrine [17] is a relevant compound to be further studied. The analysis of its capacity to kill parasite dormant forms, and identification of its main target deserve further investigation in the future.

## Supplementary information

**Additional file 1: Figure S1.** EIMS spectra of the nine compounds used in the study. **Figure S2.** 1H NMR spectra of hippeastrine. **Figure S3.** BNZ and DTX dose-response curves. Both reference drugs were included in every assay as a control of drug inhibition. Anti-*T. cruzi* assays are represented by circles while Vero and HepG2 cell toxicity assays by squares and triangles, respectively.

## Data Availability

Data supporting the conclusions of this article are included within the article and its additional file. Data and materials can be made available upon reasonable request to the authors.

## Abbreviations

BNZ: benznidazole
NFX: nifurtimox
FDA: Food and Drug Administration
GC-MS: gas chromatography-mass spectrometry
NMR: nuclear magnetic resonance
DMEM: Dulbecco’s Modified Eagle’s Medium
P-S: penicillin-streptomycin
FBS: fetal bovine serum
DMSO: dimethyl sulfoxide
PBS: phosphate-buffered saline
CPRG: chlorophenol red-β-d-galactoside
SD: standard deviation
SI: selectivity index
