# Supplementary figures and images for: Amaryllidaceae alkaloids with anti-Trypanosoma cruzi activity
Source: Parasit Vectors. 2020 Jun 10;13:299. doi: 10.1186/s13071-020-04171-6 (PMC7288428; doi:10.1186/s13071-020-04171-6)

## Additional file 1

Figure S1.

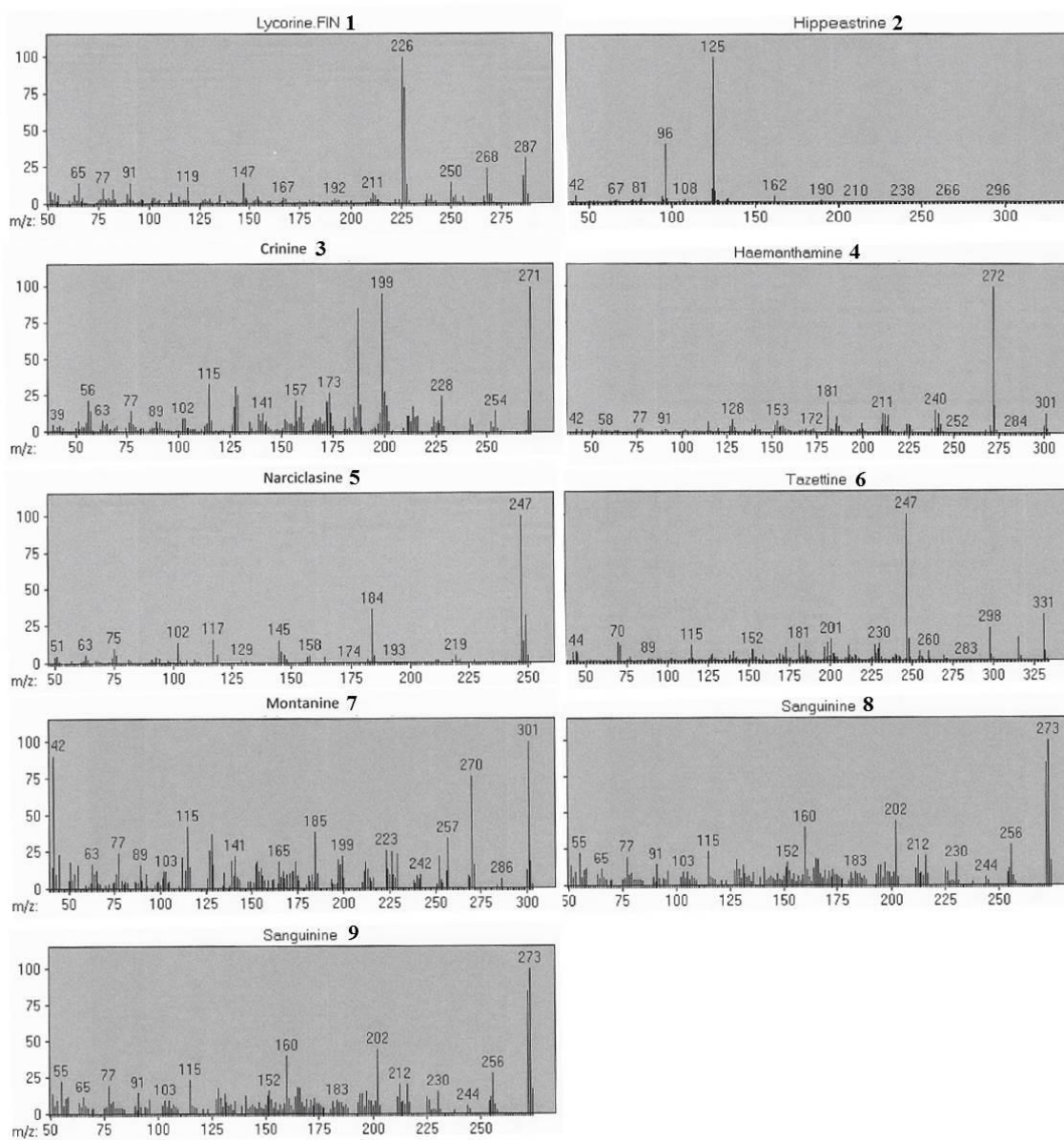

**Figure S2.**

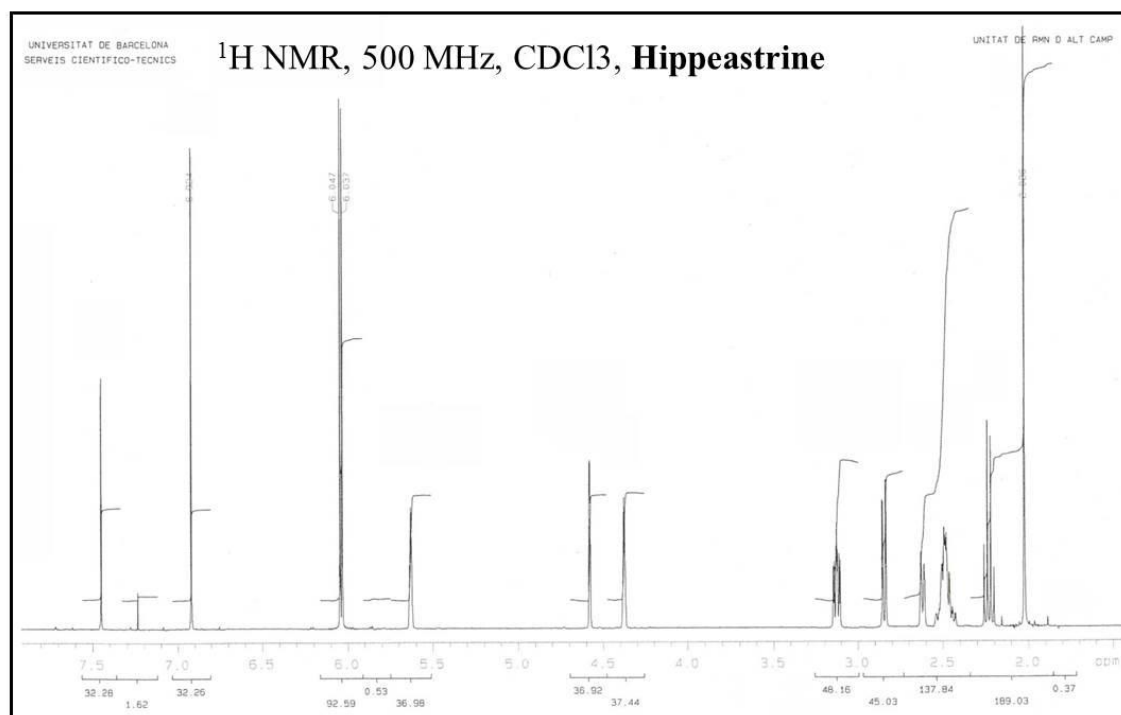

Figure S3.

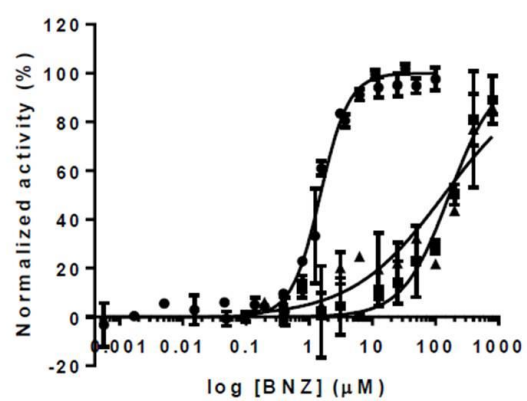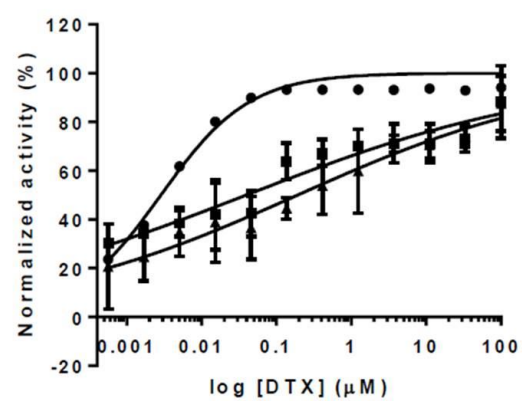

Supplement: Supplementary file 1 — Additional file 1: Figure S1. EIMS spectra of the nine compounds used in the study. Figure S2. 1H NMR spectra of hippeastrine. Figure S3. BNZ and DTX dose-response curves. Both reference drugs were included in every assay as a control of drug inhibition. Anti-T. cruzi assays are represented by circles while Vero and HepG2 cell toxicity assays by squares and triangles, respectively. [file 13071_2020_4171_MOESM1_ESM.pdf]
